# Detection of Serum Cross-Reactive Antibodies and Memory Response to SARS-CoV-2 in Prepandemic and Post–COVID-19 Convalescent Samples

**DOI:** 10.1093/infdis/jiab333

**Published:** 2021-06-23

**Authors:** Khalid Shrwani, Ravi Sharma, Madhan Krishnan, Terry Jones, Martin Mayora-Neto, Diego Cantoni, Nigel J Temperton, Susan L Dobson, Krishanthi Subramaniam, Paul S McNamara, Nigel A Cunliffe, Lance Turtle, Qibo Zhang

**Affiliations:** 1 Department of Clinical Infection, Microbiology, and Immunology, Institute of Infection, Veterinary, and Ecological Sciences, University of Liverpool, Liverpool, United Kingdom; 2 Ear Nose and Throat Department, Alder Hey Children’s Hospital, Liverpool, United Kingdom; 3 Liverpool Head and Neck Centre, University of Liverpool, Liverpool, United Kingdom; 4 Viral Pseudotype Unit, Medway School of Pharmacy, University of Kent, Chatham, United Kingdom; 5 Institute of Child Health, Alder Hey Children’s Hospital, Liverpool, United Kingdom

**Keywords:** COVID, 19, SARS, CoV, 2, common human coronavirus (HCoV)-HKU1, HCoV, NL63, serum antibody, cross, reactive immunity, immunological memory

## Abstract

**Background:**

A notable feature of coronavirus disease 2019 (COVID-19) is that children are less susceptible to severe disease. Children are known to experience more infections with endemic human coronaviruses (HCoVs) compared to adults. Little is known whether HCoV infections lead to cross-reactive anti-severe acute respiratory syndrome coronavirus 2 (SARS-CoV-2) antibodies.

**Methods:**

We investigated the presence of cross-reactive anti–SARS-CoV-2 IgG antibodies to spike 1 (S1), S1-receptor-binding domain (S1-RBD), and nucleocapsid protein (NP) by enzyme-linked immunosorbent assays, and neutralizing activity by a SARS-CoV-2 pseudotyped virus neutralization assay, in prepandemic sera collected from children (n = 50) and adults (n = 45), and compared with serum samples from convalescent COVID-19 patients (n = 16).

**Results:**

A significant proportion of children (up to 40%) had detectable cross-reactive antibodies to SARS-CoV-2 S1, S1-RBD, and NP antigens, and the anti-S1 and anti–S1-RBD antibody levels correlated with anti–HCoV-HKU1 and anti–HCoV-OC43 S1 antibody titers in prepandemic samples (*P* < .001). There were marked increases of anti–HCoV-HKU1 and - OC43 S1 (but not anti-NL63 and -229E S1-RBD) antibody titers in serum samples from convalescent COVID-19 patients (*P *< .001), indicating an activation of cross-reactive immunological memory to β-coronavirus spike.

**Conclusions:**

We demonstrated cross-reactive anti–SARS-CoV-2 antibodies in prepandemic serum samples from children and young adults. Promoting this cross-reactive immunity and memory response derived from common HCoV may be an effective strategy against SARS-COV-2 and future novel coronaviruses.

## Teaser

We demonstrated preexisting cross-reactive anti–SARS-CoV-2 antibodies in prepandemic serum samples from children and young adults, which were likely derived from previous infection with common endemic β-coronaviruses. Promoting this cross-reactive immunity may be an effective strategy against SARS-COV-2 and future novel coronaviruses.

Severe acute respiratory syndrome coronavirus 2 (SARS-CoV-2), which causes coronavirus disease 2019 (COVID-19), belongs to the genus *Betacoronavirus*. As of 14 June 2021, the pandemic had accounted for over 176 million infections and caused more than 3.8 million deaths worldwide. While most people are susceptible to SARS-CoV-2 infection, there are considerable differences in disease susceptibility among individuals. Patients with COVID-19 exhibit variable disease severity, with older age groups typically experiencing more severe disease and worse outcomes. However, many individuals, particularly younger people including children, exhibit mild or no symptoms following infection with SARS-CoV-2. The immunological mechanisms underpinning these age-dependent differences are not understood. There has been much interest to determine whether there is any preexisting cross-reactive immunity to SARS-CoV-2 due to prior exposures to endemic human common coronavirus (HCoV). These include the α-HCoV (HCoV-NL63 and HCoV-229E) and β-HCoV (HCoV-HKU1 and HCoV-OC43), which cause upper respiratory infections such as common colds [1, 2]. Recent reports have suggested the presence of cross-reactive T cells [3–5] and serum antibodies [6, 7], although the protective function of the cross-reactive T cells is uncertain [8].

We have investigated preexisting cross-reactive antibody levels to SARS-CoV-2, including immunoglobulin G (IgG) antibodies to the spike 1 (S1), S1 receptor-binding domain (S1-RBD), and nucleocapsid protein (NP) antigens, in prepandemic serum samples collected from children and adults, and compared these to the antibody levels in serum samples from convalescent COVID-19 patients. We have shown that a significant proportion of children (up to 40%) had detectable preexisting cross-reactive antibodies to SARS-CoV-2, which correlated with anti-HCoV-HKU1 S-RBD and anti-OC43 S1 antibody titers in the prepandemic samples. In addition, we have demonstrated a marked enhancement of anti-HCoV-HKU1 S-RBD and anti-OC43 S1 antibody titers in serum samples from convalescent COVID-19 patients, indicating an activation of cross-reactive immunological memory response to conserved β-coronavirus spike antigens.

## METHODS

### Patients and Samples

Convalescent serum samples from adult patients (aged 30–71 years) with polymerase chain reaction (PCR)-confirmed COVID-19 were collected between April 2020 and August 2020. These patients presented mild to moderate clinical manifestations of SARS-CoV-2 infection but did not require hospitalization. Healthy volunteers of similar age (30–72 years) with prepandemic samples (May 2014 to May 2018) served as controls. In addition, to investigate the presence of preexisting cross-reactive antibodies in a younger-age population, prepandemic serum samples obtained from immune-competent children and adults (aged 1.5–56 years) undergoing elective adenotonsillectomy for upper airway obstruction were studied. These samples were collected between May 2016 and May 2019 as part of an in vitro study on human cellular immunity to novel influenza vaccines [9]. Individuals with known immunocompromising conditions were excluded. The study was ethically approved by South East Scotland Research Ethics Committee (14/SS/1058, patients) and Liverpool Central Research Ethics Committee (16/NW/0170, healthy donors), and written informed consent was obtained from parents or individuals.

### Antigens and Measurement of CoV Antigen-Specific Antibodies by Enzyme-Linked Immunosorbent Assay

Antigen-specific IgG antibodies to SARS-CoV-2 S1, S1-RBD, and NP antigens, and antibodies to HCoV-HKU1 S1-RBD, HCoV-NL63 S1-RBD, HCoV-OC43 S1, and HCoV-229E S1-RBD were analyzed using a standard enzyme-linked immunosorbent assay (ELISA) procedure as previously described [10]. Briefly, ELISA plates were coated with recombinant SARS-CoV-2 S1 (Native Antigen/LGC), S1-RBD (BEIR/American Type Culture Collection), NP (Stratech), HCoV-HKU1 S1-RBD and HCoV-NL63 S1-RBD (R & D Systems), and HCoV-OC43 S1 and HCoV-229E S1-RBD proteins (Native Antigen/LGC) at appropriate concentrations following optimization, and incubated at 4°C overnight. After blocking with phosphate-buffered saline (PBS) buffer containing 10% FBS for 1.5 hours, serum samples were added at optimized dilutions and incubated for 2 hours. In the meantime, the reference serum sample was also incubated at a serial 10-fold dilution from 1:100 to 1:12800. Alkaline phosphatase-conjugated anti-human IgG was added and incubated for 1.5 hours. This was followed by addition of the substrate *p*-nitrophenyl phosphate, and optical density was measured at 30 minutes using a plate reader (Labtech). Antibody positivity cutoff values were determined based on mean plus 2 standard deviations (SD) of optical densities of the prepandemic control samples from healthy adult volunteers.

As there were no reference standards for the measurement of different CoV antibodies, individual serum samples containing high antibody titers to different CoV antigens were used as individual reference sera, respectively. A convalescent serum sample from a subject with confirmed COVID-19 infection and 2 other samples with high titers of anti-HCoV (HKU1, NL63, OC43, and 229E) antibodies were used as reference standards for anti–SARS-CoV-2 and anti-HCoV antibodies, respectively. Following a standard ELISA procedure with the use of serial dilutions of individual reference sera, arbitrary antibody units were assigned to each reference standard as the reciprocal of the dilution factor giving an OD 405 nm = 1. Once the antibody units were assigned for the reference sera, they were used on each ELISA plate to create a standard curve for analysis of all samples [11]. Samples with undetectable antibody titer for each assay were assigned a value of half of the lower detection limit.

The specificity of the ELISAs was confirmed by antigen-specific inhibition assays using methods as described previously [12] by adsorption with individual recombinant antigens. These ELISA assays measure antibody titers of all the antibodies binding to the coating CoV antigen and therefore may include both neutralizing and non-neutralizing antibodies.

### SARS-CoV-2 Pseudotyped Virus Neutralization Assay

Production of SARS-CoV-2 pseudotype virus (PV) was carried out as described previously [13]. Briefly, HEK 293T cells were seeded at 50% confluence for next-day transfection. Plasmids used for transfection were 1000 ng SARS-CoV-2 spike (pcDNA3.1), 1500 ng lentiviral vector expressing firefly luciferase pCSFLW, and 1000 ng second-generation lentiviral packaging plasmid p8.91 expressing gag, pol, and rev. They were mixed in 200 µL Optimem for 5 minutes, followed by addition of FuGENE HD at a ratio of 1:3 (DNA:FuGENE HD), and incubated for 15 minutes at room temperature before adding the transfection mix to the cells. PVs were harvested after 48 hours by filtration using a 0.45-µm cellulose acetate filter.

Target HEK293T cells were transfected using plasmids expressing ACE2 (pcDNA3.1+) and TRSSMP2 (pCAGGS). For titration, 100 µL of harvested PV was added in the first row of a 96-white-well plate, followed by 50 µL of Dulbecco’s Modified Eagle’s Medium (DMEM) to all other wells. A 2-fold serial dilution was carried out. Target cells were then added at a density of 10 000 cells per well and incubated for 48 hours. Bright-Glo (Promega) was subsequently added, incubated for 5 minutes, and then luciferase expression was quantified using a GloMax Navigator microplate luminometer. For neutralization, patient sera were serially diluted 2-fold in complete DMEM starting at a 1:10 dilution in a 96-white-well plate and mixed with SARS-CoV-2 PVs for 1 hour at 37°C. Target HEK293T cells were then added (10 000 cells/well), and plates were incubated for 48 hours prior to assaying luciferase expression levels. Data analysis to derive 50% inhibitory concentration (IC_50_) was carried out using GraphPad Prism 8 software.

### Statistical Analysis

Differences in antibody titers between different groups were analyzed by Student *t* test. Association between 2 factors was analyzed by Pearson correlation. A *P* value of <.05 was considered statistically significant.

## RESULTS

### **Detection of Anti**–**SARS-CoV-2 Antibodies to Spike 1, S1-RBD, and NP Antigens in Convalescent COVID-19 Patients**

**Figure 1.**
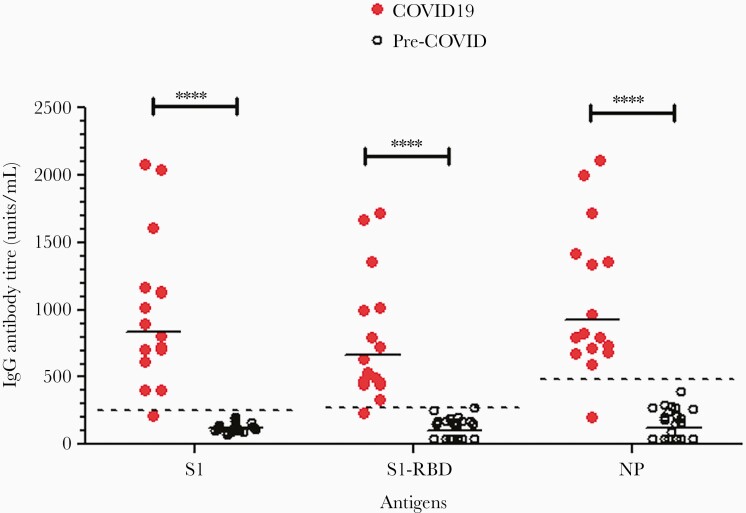
*A*, Detection of anti–SARS-CoV-2 spike 1, S1-RBD, and NP antibodies in convalescent sera from COVID-19 patients (n = 16) as compared to age-matched prepandemic healthy control samples (n = 22). ****P < .0001. Horizontal bars represent geometric mean antibody titers in each group. Dashed lines indicate the antibody positivity cutoff for anti-S1, S1-RBD, and NP IgG antibodies. *B*, Correlations between the antigen-specific anti–SARS-CoV-2 IgG antibodies to S1, S-RBD, and NP antigens in the convalescent samples (Pearson *r* = 0.85, 0.86, and 0.78, respectively). Abbreviations: COVID-19, coronavirus disease 2019; IgG, immunoglobulin G; NP, nucleocapsid protein; RBD, receptor-binding domain; S, spike; SARS-CoV-2, severe acute respiratory syndrome coronavirus 2.

Anti–SARS-CoV-2 IgG antibodies to the S1, S1-RBD, and NP antigens in serum samples from convalescent COVID-19 patients (aged 30–71 years, n = 16) were analyzed by antigen-specific ELISA. The antibody titers in COVID-19 patients were compared to the prepandemic age-matched healthy control samples (age range, 30–72 years, n = 22; Figure 1A). The majority of antibody titers in COVID-19 patient samples were significantly higher than the mean background level based on prepandemic healthy control samples (*P *< .0001). Furthermore, antigen-specific IgG titers to S1, S1-RBD, and NP antigens in the convalescent samples correlated well (*r* = 0.85, 0.86, and 0.78 respectively; Figure 1B).

### Detection of Cross-Reactive Antibodies to SARS-CoV-2 in Prepandemic Samples From Children and Adults

**Figure 2.**
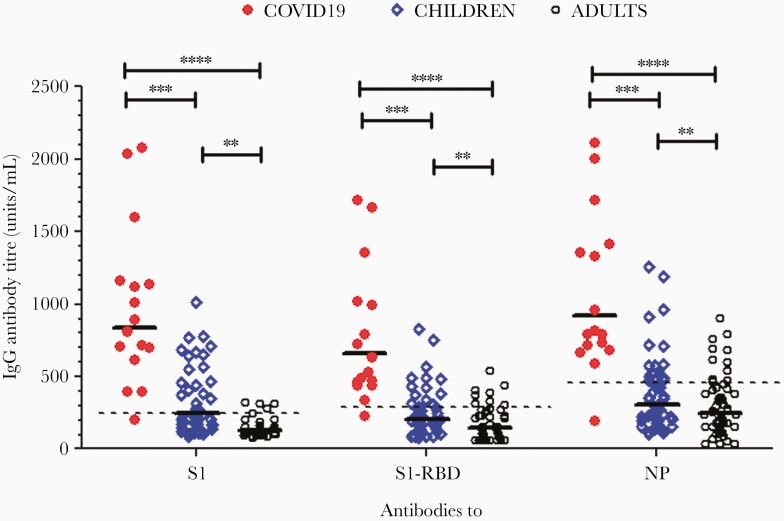
Detection of preexisting cross-reactive antibodies to SARS-CoV-2 in prepandemic samples from children (n = 50) and adults (n = 45) undergoing elective adenotonsillectomy due to upper airway obstruction, as compared with convalescent COVID-19 patient samples (n = 16). Horizontal bars represent geometric mean antibody titers of antibody in each group, and dashed lines indicate the antibody positivity cutoff for anti-S1, S-RBD, and NP IgG antibodies. ** *P *< .01, ***P < .001, ****P < .0001. Abbreviations: COVID-19, coronavirus disease 2019; IgG, immunoglobulin G; NP, nucleocapsid protein; RBD, receptor-binding domain; S, spike; SARS-CoV-2, severe acute respiratory syndrome coronavirus 2.

To test the hypothesis that there were preexisting cross-reactive antibodies to SARS-CoV-2 in children and adults, prepandemic serum samples (collected May 2016 to May 2019) from immune-competent children (n = 50, aged 2–16 years) and adults (n = 45, aged 17–50 years) undergoing elective adenotonsillectomy were studied. As shown in Figure 2, a significantly higher proportion of prepandemic samples from these children (34%–40%), as compared to adults (8%–12%), had positive antibody titers for anti–SARS-COV-2 S1, S1-RBD, and NP IgG antibodies (*P* < .001).

**Figure 3.**
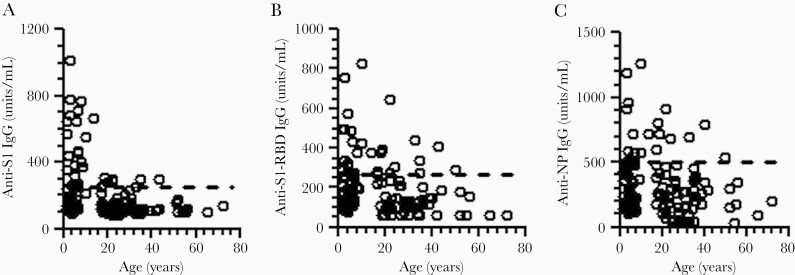
Association between the cross-reactive anti–SARS-CoV-2 S1 (*A*), S-RBD (B), and NP (*C*) antibody titers and age in prepandemic serum samples from children and adults. Dashed lines indicate the antibody positivity cutoff for anti-S1, S-RBD, and NP IgG antibodies. Abbreviations: IgG, immunoglobulin G; NP, nucleocapsid protein; RBD, receptor-binding domain; S, spike; SARS-CoV-2, severe acute respiratory syndrome coronavirus 2.

We further studied the relationship between the cross-reactive anti–SARS-CoV-2 antibody levels and age in the prepandemic samples. There was an inverse relationship between the antibody levels and age, with younger people and particularly children having higher preexisting cross-reactive antibodies to SARS-CoV-2 than older individuals (Figure 3A–3C).

### Relationship between Cross-Reactive Antibodies to SARS-CoV-2 and Antibodies to HCoV in Prepandemic Samples

**Figure 4.**
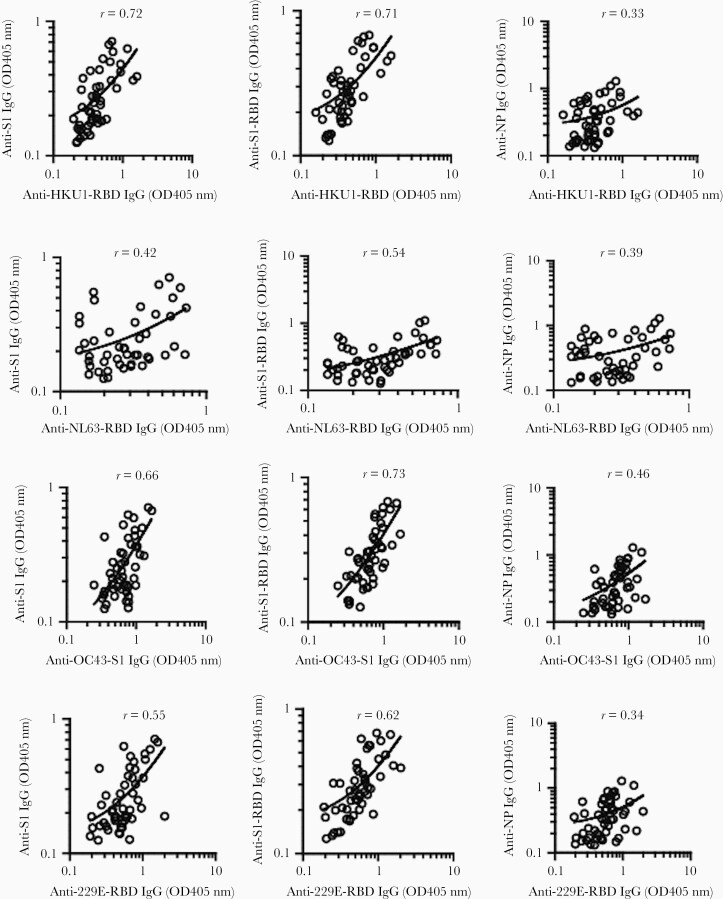
Correlations (Pearson *r*) between preexisting cross-reactive anti–SARS-CoV-2 IgG antibodies (to S1, S-RBD, and NP) and anti-HKU1 S-RBD, anti-NL63 S-RBD, anti-OC43 S1, and anti-229E S-RBD antibodies in prepandemic samples from children and adults (n = 110). Antibody titers expressed as original OD values were preferred in this correlation analysis as many samples have undetectable antibody titers (units/mL). Abbreviations: IgG, immunoglobulin G; NP, nucleocapsid protein; OD, optical density; RBD, receptor-binding domain; S, spike; SARS-CoV-2, severe acute respiratory syndrome coronavirus 2.

To investigate whether preexisting antibodies to SARS-CoV-2 were associated with cross-reactive immunity to HCoVs, we analyzed HCoV spike-specific antibodies to NL63 and 229E (α-HCoV), and HKU1 and OC43 (β-HCoV), and their correlations with cross-reactive antibody titers to SARS-CoV-2 in prepandemic samples. There were high correlations between anti–SARS-CoV-2 antibodies (to S1, S1-RBD) and anti-HKU1 RBD antibody titers (*r *= 0.72 and 0.71, respectively; *P *< .0001), and between anti–SARS-CoV-2 antibodies and anti-OC43 S1 antibodies (*r* = 0.66 and 0.73, respectively; *P *< .0001). There were only modest correlations between anti–SARS-CoV-2 antibodies (to S1, S1-RBD) and anti–NL63-RBD antibody titers (*r* = 0.43 and 0.53, respectively; *P *< .01), and between anti–SARS-CoV-2 antibodies and anti-229E RBD antibody titers (*r* = 0.55 and 0.62, respectively; *P *< .001; Figure 4).

**Figure 5.**
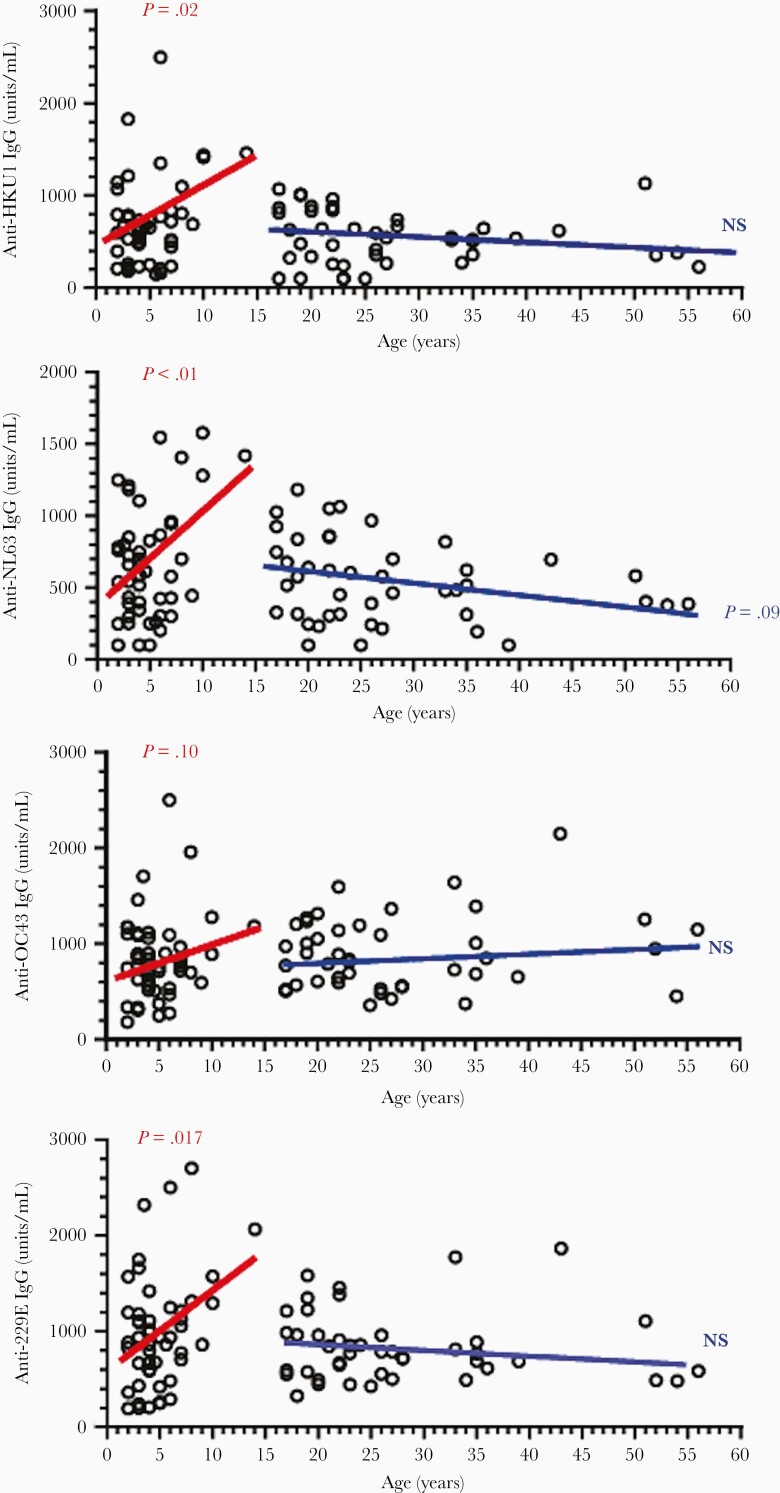
Correlations between age and anti-HCoV (HKU1 S1-RBD, NL63 S1-RBD, OC43 S1, and 229E S1-RBD) IgG antibody titers in prepandemic samples from children and adults undergoing adeotonsillectomy. The regression lines and *P* values are shown for children (red) and adults (blue). Abbreviations: HCoV, human coronavirus; IgG, immunoglobulin G; NS, not significant; RBD, receptor-binding domain; S, spike.

Next, we analyzed the relationship between age and anti-HCoV (HKU1, NL63, OC43, and 229E) S1 antibody titers in prepandemic samples from children and adults undergoing elective adeotonsillectomy. In children (aged 1–14 years), there was a significant correlation between age and anti-HCoV (HKU1, NL63, and 229E) antibody titers, although not for anti-OC43 IgG titers. In adults, however, anti-HCoV antibody titers remained relatively static with rising age (Figure 5).

Furthermore, we performed antigen inhibition assay to assess the extent of cross-reactivity between anti–SARS-CoV-2 S1 and anti-HKU1 S1-RBD antibodies. At 10 μg/mL antigen concentration, prior adsorption of sera with SARS-2 S1 and HKU1 S1-RBD antigens inhibited the anti–SARS-CoV-2 S1 antibody levels by a mean of 50% and 35%, respectively, compared to a mean inhibition of 16% following NL63 S1-RBD adsorption. Similarly, for anti-HKU1 S1-RBD antibody titers, the mean inhibitions following adsorption by HKU1 S1-RBD, SARS-CoV-2 S1, and NL63 S1-RBD were 51%, 32%, and 6%,respectively. For anti-NL63 S-RBD antibody titers, no significant inhibition was seen following adsorption with the HKU1 and SARS-CoV-2 S1 antigens (Supplementary Figure 1).

### Enhancement of Anti-HKU1 Antibody Levels Following COVID-19 Reveals Cross-Reactive Memory

**Figure 6.**
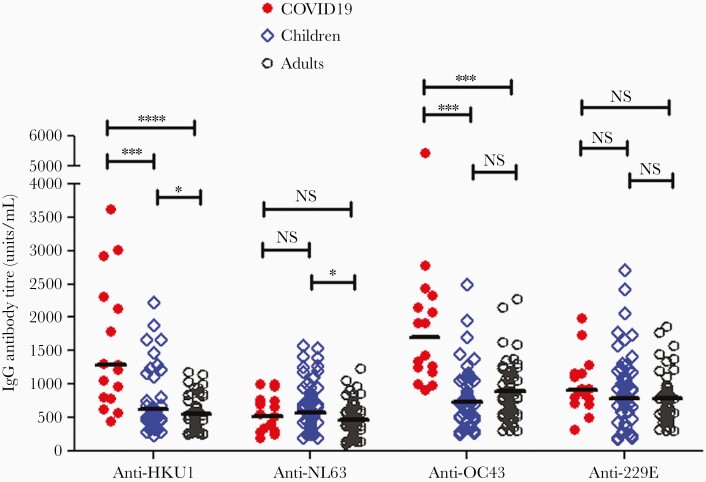
Enhancement of anti-HKU1 S1-RBD and anti-OC43 S1 antibody levels following COVID-19 suggests cross-reactive antibody memory response. Anti-HCoV (HKU1 S1-RBD, NL63 S1-RBD, OC43 S1, and 229E S1-RBD) antibody titers were compared between post–COVID-19 (n = 16) and prepandemic samples from children (n = 50) and adults (n = 67). Horizontal bars represent geometric mean antibody titer in each group. *P < .05, ***P < .001, ****P < .0001. Abbreviations: COVID-19, coronavirus disease 2019; HCoV, human coronavirus; IgG, immunoglobulin G; NS, not significant; RBD, receptor-binding domain; S, spike.

To investigate whether SARS-CoV-2 infection could activate a cross-reactive memory response to HCoVs, we compared the anti-HCoV (HKU1, NL63, OC43, and 229E) antibody titers between post–COVID-19 and prepandemic samples. There were marked increases in anti-HKU1 S1-RBD and anti-OC43 S1 IgG responses in convalescent COVID-19 samples, as compared to prepandemic samples of children and adults, whereas no enhancement in anti-NL63 and anti-229E S1-RBD IgG responses was observed (Figure 6). This indicated that SARS-CoV-2 infection boosted the antibody responses cross-reactive to HKU1 and OC43 S1 antigens (β-HCoV). Furthermore, we observed in the prepandemic samples that children had a higher level of anti-HCoV-HKU1 and anti-NL63 S1-RBD antibody levels than in adults, although no difference was seen between children and adults in anti-OC43 and anti-229E antibody levels (*P *< .01; Figure 6).

### **Detection of Anti**–**SARS-CoV-2 Neutralizing Antibody in Prepandemic Samples**

**Figure 7.**
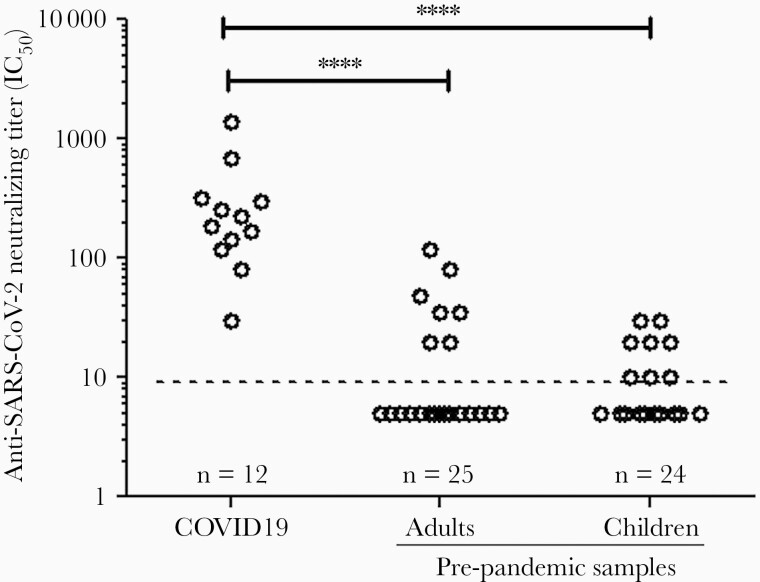
Detection of anti–SARS-CoV-2 neutralizing antibody titers by pseudotyped SARS-COV-2 virus in convalescent COVID-19 and prepandemic samples from children and adults. Dashed horizontal line represents the detection limit of neutralizing activity. Abbreviations: COVID-19, coronavirus disease 2019; IC_50_, 50% inhibitory concentration; SARS-CoV-2, severe acute respiratory syndrome coronavirus 2.

Because prepandemic samples, particularly those from children and young adults, demonstrated cross-reactive anti–SARS-CoV-2 IgG antibodies, we next tested these samples for anti–SARS-CoV-2 neutralizing activity. Using a SARS-COV-2 pseudotype virus neutralization assay, we showed that similar small proportions of the prepandemic samples from children (7/25, 29%) and adults (7/24, 28%) had detectable neutralizing activity (Figure 7). These neutralizing samples were generally associated with a higher level of cross-reactive antibody titers to SARS-CoV-2 S1 and S1-RBD. Overall, the neutralizing titers in the prepandemic samples were modest and lower than most of the convalescent COVID-19 patient samples (Figure 7). There was a significant correlation between the neutralizing titers and anti–SARS-CoV-2 S1-RBD antibody levels in convalescent COVID-19 samples (*r* = 0.61, *P *< .0001, data not shown).

## Discussion

We demonstrated the presence of cross-reactive anti–SARS-CoV-2 S1, S1-RBD and NP antibodies in prepandemic serum, predominantly in the pediatric and young adult samples, and there was a corresponding good relationship between the cross-reactive anti–SARS-CoV-2 spike and anti–HCoV-HKU1 and -OC43 S1 antibodies. Furthermore, we demonstrated a marked enhancement of anti–HCoV-HKU1 and -OC43 S1 antibody responses in convalescent COVID-19 patient samples, suggesting the activation of cross-reactive immune memory.

It has been speculated that common endemic HCoVs may, to some extent, induce cross-reactive immunity to SARS-CoV-2, although current evidence is limited. Recent reports have suggested the presence of preexisting T-cell immunity to SARS-CoV-2 in prepandemic samples [3–5]. However, these cells seem to be of low avidity compared with those that develop after SARS-CoV-2 infection, and may not participate in immunity very effectively [8]. With regard to potential cross-reactive antibody immunity, it has been reported that anti–SARS-CoV-2 spike antibodies in the circulation are uncommon in individuals not exposed to SARS-CoV-2, and cross-reactive neutralizing antibodies appear to be rare [14]. However, a recent report suggested the presence of preexisting cross-reactive antibodies to SARS-CoV-2 spike in young people including children, mostly against the S2 domain [7].

In this study, we demonstrated the presence of cross-reactive anti–SARS-CoV-2 antibodies (to S1, S1-RBD, and NP) in a proportion of prepandemic samples from children and young adults who underwent routine elective surgery (adenotonsillectomy). On the other hand, the presence of cross-reactive antibodies appeared to be rare in the prepandemic samples donated from healthy adult volunteers (aged 30–72 years). There is a possibility that patients who undergo adenotonsillectomy may have an increased exposure to cross-reactive HCoVs as compared to the general healthy population, as some of the patients aged 20–40 years also showed detectable cross-reactive antibody level. The clear age-associated decrease in the antibody levels in this patient population supports age as an independent determinant. These findings would provide support to the hypothesis that young people, particularly children, may develop cross-reactive antibodies to coronaviruses because of frequent exposure to HCoV.

One of the most intriguing features of the COVID-19 pandemic is that young people, and particularly children, are less susceptible to severe COVID-19 disease as compared to adults [15, 16], although lower expression of the virus receptor (angiotensin-converting enzyme 2, ACE2) in pediatric nasal epithelium was reported [17]. It has been shown in a number of studies that children experience more infections with common endemic HCoVs, including the α- and β-HCoV, than adults [18–20]. Our results showed that in children anti-HCoV antibody titers (anti-HKU1, -NL63, and -229E) increase with age from 1 to 14 years. Marginally lower antibody titers are then observed in adults, although no significant change with age is seen among adults. This is in agreement with the data reviewed by Huang et al [21] that seroprevalence rises sharply in childhood, with little or no change by age among adults. Our data in this cohort demonstrated that, overall, children showed higher levels than adults in anti-HKU1 and anti-NL63 antibodies, but no difference in anti-OC43 and anti-229E antibodies. It is therefore plausible that immune responses induced by frequent HCoV infections during childhood lead to the formation of immunological memory cells, and that cross-reactive memory T and B cells would be boosted following subsequent exposure/infection leading to detectable cross-reactive CoV antibody. We demonstrated an inverse relationship in the cross-reactive anti–SARS-CoV-2 antibody level with age, consistent with the hypothesis that the presence of cross-reactive antibodies is more common in young people, particularly children, compared with older adults. Consistent with our finding, a recent study also reported detection of preexisting anti-spike antibodies by a flow-cytometry-based method in prepandemic samples that were particularly prevalent in children and adolescents, although they appeared to target mainly the S2 subunit [7]. Furthermore, a study on pediatric and adult COVID-19 patients also demonstrated distinct antibody responses in children and adults, and in pediatric patients aged 3 to 18 years, there was an age-dependent decrease in both anti–SARS-CoV-2 spike IgG antibody level and neutralizing activity [22].

We further analyzed the anti-spike antibodies to the common HCoV including HKU1, OC43, NL63, and 229E, in the prepandemic samples, and showed a good correlation between the cross-reactive anti–SARS-CoV-2 spike antibodies (to S1 and S1-RBD) and anti-HKU1 S-RBD and anti-OC43 S1 antibodies, but only a weak correlation with anti-NL63 and anti-229E S-RBD antibodies. This suggests that HCoV-HUK1 and -OC43 (both β-HCoV) induce anti-spike antibodies cross-reactive to SARS-CoV-2 spike due to their antigenic similarity. Our results also suggest that the cross-reactive anti-spike IgG antibodies also target the S1 domain in addition to the S2 domain, as suggested by Ng et al [7]. Interestingly, when we compared anti-HKU1 and -OC43 S1 antibody levels between convalescent COVID-19 samples and prepandemic samples, there were marked increases of both anti-HKU1 and -OC43 S1 antibody titers in COVID-19 patients as compared to prepandemic samples from both children and adults. In contrast, there was no increase in anti-NL63 and -229E spike RBD antibody levels in COVID-19 patients. These results are consistent with the recent report by Anderson et al that prepandemic samples with detectable cross-reactive SARS-CoV-2 antibodies had higher anti-OC43 S protein antibody levels, and SARS-CoV-2 infection boosted antibody levels to HCoV-OC43 but not HCoV-NL63 among COVID-19 patients [23].

These results further support the notion that there is cross-reactive immune recognition between β-HCoV (HKU1, OC43) and SARS-CoV-2, and suggest the possibility that SARS-CoV-2 infection boosted the cross-reactive memory anti-spike antibody response primed by previous β-HCoV (HKU1 and OC43) exposure. In principle, SARS-CoV-2 S cross-reactive memory B cells could be preexisting in the COVID-19 donors and show cross-reactivity with SARS-CoV-2 or originate from SARS-CoV-2 infection and show cross-reactivity with HCoV S protein. The study by Song et al analyzing memory B cells from convalescent COVID-19 patients supports that elevated antibody levels to HCoV-HKU1 S-protein in COVID-19 donors is consistent with the former, as SARS-CoV-2 activates B cells expressing preexisting HCoV-HKU1 S-specific B cell receptors to secrete the corresponding antibodies [6]. These findings therefore provide evidence of preexisting cross-reactive memory B cells, due to previous β-HCoV (HKU1 and OC43) exposure, and a recall of cross-reactive antibodies upon SARS-CoV-2 infection.

Antibodies with neutralizing activity are considered important in protection against SARS-CoV-2 infection. Many studies demonstrated a close correlation between anti–SARS-CoV-2 spike IgG antibody level and neutralizing activity [22, 24], which was also shown in this study, suggesting a critical role of anti-spike antibody in virus neutralization. When we analyzed the virus neutralizing activity in the prepandemic samples using a SARS-CoV-2 pseudotyped neutralizing assay, there were a small proportion of child and adult samples showing detectable neutralizing activity, although the neutralizing titers were generally lower than that observed in convalescent COVID-19 sera. Nevertheless, some prepandemic samples did show neutralizing antibody titers that overlapped with convalescent COVID-19 sera with lower antibody titers. The finding that many children had preexisting cross-reactive anti–SARS-CoV-2 IgG antibodies but only modest or undetectable neutralizing activity is consistent with a previous report showing that cross-reactive antigen binding was common whereas cross-neutralization was rare between SARS-CoV-2 and SARS-CoV [25].

The protective levels of neutralizing activity or the IgG antibodies against infection or severe disease are as yet unknown, although experiment in nonhuman primates suggested relatively low antibody titers were sufficient for protection against SARS-CoV-2 [26]. It is plausible that higher neutralizing activity is associated with protection against new infection (eg, following vaccination [27]), whereas antibodies can also provide protection against severe disease via mechanisms other than neutralization, such as antibody-dependent cell-mediated cytotoxicity [28]. A study suggested that recent endemic HCoV infections are not associated with decreased SARS-CoV-2 infections but are associated with reduced severity of COVID-19 [29]. However, in the study by Anderson et al, no protection was shown against severe COVID-19 by cross-reactive HCoV antibodies, although larger cohorts of patients with different disease severities will be required to determine whether preexisting cross-reactive antibodies are associated with protection either against infection or severe COVID-19 [23].

On the other hand, a number of studies demonstrated the presence of preexisting cross-reactive T cells in prepandemic samples [3–5]. In the context that SARS-CoV-2 appeared to share predominantly non-neutralizing antibody epitopes with previously circulating HCoVs, non-neutralizing antibody responses or cross-reactive T cells may play a role in protection against severe COVID-19 in populations such as children, and further studies are warranted in this area.

In conclusion, our findings of higher cross-reactive antibody levels in younger individuals may help explain why children are relatively protected against severe COVID-19 disease. The mechanism of action, and protective levels of IgG or neutralizing antibodies against infection or severe disease warrants further investigation. The results suggest significant cross-reactive immune recognition between β-HCoV (HKU1, OC43) and SARS-CoV-2, and activation of cross-reactive anti-spike antibody memory response following SARS-CoV-2 infection. Promoting common HCoV primed cross-reactive immunity and memory may be an important and effective strategy against SARS-CoV-2 and future outbreaks or pandemics by novel coronaviruses. The detection of cross-reactive immunity to SARS-COV-2 in children may have important implications in the potential impact of COVID-19 vaccination in children including long-term immunity to SARS-CoV-2, which should be included in future studies. Our study only examined serum antibodies in 1 cohort of patients and thus is limited in scope. Future larger studies that include investigation on the potential protection by the cross-reactive antibodies, and on memory B and T cells, will add invaluable information on disease prevention.

## Supplementary Data

Supplementary materials are available at *The Journal of Infectious Diseases* online. Consisting of data provided by the authors to benefit the reader, the posted materials are not copyedited and are the sole responsibility of the authors, so questions or comments should be addressed to the corresponding author.

jiab333_suppl_Supplementary_Figure_S1

## Notes

***Acknowledgments.*** We thank the patients who took part in the study and the theatre staff in the Liverpool Children’s Hospital and Aintree University Hospital for helping the collection of samples.

***Author contributions.*** K. S., P. M., N. A., L. T., and Q. Z. conceived and designed the experiments. K. S., M. M. D., D. C., N. A. T., and Q. Z. performed the experiments and analysis. R. S., M. K., T. J., S. D., and K. S. contributed clinical samples. All authors contributed to the writing of the manuscript.

***Disclaimer***. The funders had no role in study design, data collection, and interpretation.

***Financial support***. This work was support by UK Immunising pregnant women and infants network (IMPRINT) (grant to Q. Z.) and BactiVac Vaccine Networks (grant number BVNCP2-04 to Q. Z.); the Center for Disease Prevention and Control of Saudi Arabia (studentship to K. S.); the Wellcome Trust (grant numbers GB-CHC-210183 to N. T. and 205228/Z/16/Z fellowship to L. T.); Medical Research Council (grant number MC_PC_19060 to N. T.); National Institute for Health Research (NIHR) (grant number MC_PC_20016 to N. T); and the National Institute for Health Research (NIHR) Health Protection Research Unit in Emerging and Zoonotic Infections (grant number NIHR200907 to L. T. at University of Liverpool in partnership with Public Health England, in collaboration with Liverpool School of Tropical Medicine and University of Oxford).

***Potential conflicts of interest.*** All authors: No reported conflicts of interest. All authors have submitted the ICMJE Form for Disclosure of Potential Conflicts of Interest. Conflicts that the editors consider relevant to the content of the manuscript have been disclosed.
