# Supplementary figures and images for: Detection of Serum Cross-Reactive Antibodies and Memory Response to SARS-CoV-2 in Prepandemic and Post–COVID-19 Convalescent Samples
Source: J Infect Dis. 2021 Jun 23;224(8):1305–15. doi: 10.1093/infdis/jiab333 (PMC8557674; doi:10.1093/infdis/jiab333)

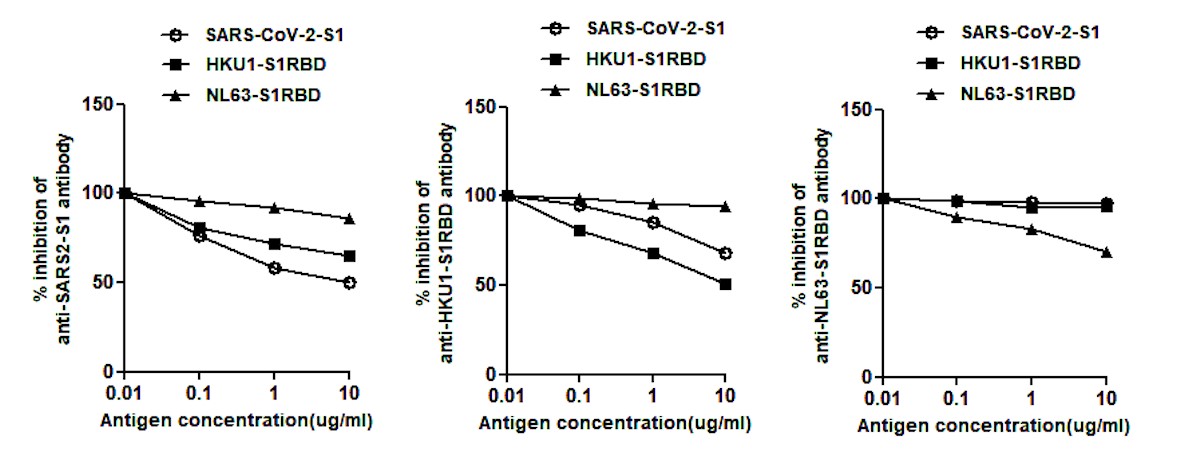

Supplement: jiab333_suppl_Supplementary_Figure_S1 [file jiab333_suppl_supplementary_figure_s1.jpeg]
